# The Relative Dangers of Anæsthesia by Chloroform and Ether—Statistics of 209,893 Cases

**Published:** 1870-05

**Authors:** E. Andrews

**Affiliations:** Prof. of Principles and Practice of Surgery, in Chicago Medical College


### CHICAGO MEDICAL EXAMINER.

N. S. DAVIS, M.D., Editor.

VOL. XI. MAY, 1870. NO. 5.

### Original Contributions.

ARTICLE XIII.

### THE RELATIVE DANGERS OF ANÆSTHESIA BY CHLOROFORM AND ETHER—STATISTICS OF 209,893 CASES.

By E. ANDREWS, A.M., M.D., Prof. of Principles and Practice of Surgery, in Chicago Medical College.

The determination of the actual rates of danger in the use of our principal anæsthetics is a desideratum long felt by surgeons, but one which, owing to the difficulties of the undertaking, has generally been despaired of. M.M. Perrin and Lallemand, of France, in their elaborate work upon anæsthesia, pronounce the accomplishment of the task impossible. They remark, that to obtain the result, it would be necessary to "compare the number of the fatal cases with the approximate number of etherizations, so as to deduce the proportion of deaths. That last term of the comparison is wanting, and we hesitate not to say will always be wanting." (*Traite d'Anesthesie*, pp. 235, 236.)

Other surgeons seem to have been equally hopeless of success in this matter, so that our best works on anæsthesia are often perfectly silent, on the important point of the relative dangers of the different articles. Yet this is the very thing on which the surgeon most needs light, at the present time. It is well known that chloroform is by far the most convenient article for use, and, therefore, always to be preferred, if equally safe; but if it is materially more dangerous than ether. the conscientious surgeon will choose the latter, for the sake of the safety of his patient. This is, therefore, a question of tremendous magnitude, possibly involving thousands of lives in its decision, hence the surgical profession will not quietly acquiesce in the opinion that its solution is impossible.

A three years' investigation has shown that this apparent impossibility may be overcome, and that although mathematical precision may be unattainable, yet a statistical approximation, sufficient for all practical purposes, may be established. Although we can ascertain neither the whole number of anæsthesias which have taken place in the world, nor the whole number of resulting deaths, yet we can obtain from hospitals and private records numerous masses of statistics, each of which gives a great number of anæsthesias, and the exact number of deaths pertaining to it; and by adding many such masses together, we can obtain cases enough to establish the ratio of danger. My figures thus obtained give us 92,815 cases of anæsthesia by ether, and 117,078 by chloroform, with the resulting deaths from each.

Up to the present time, the attempts to determine the ratio of danger have been as follows:—In 1859, Dr. John Chapman, of England, made a rough estimate, that chloroform had been given in all the hospitals of the world about 1,200,000 times; at the same time he gathered from books and journals 74 recorded deaths. He assumed that this represented the entire number killed by the anæsthetic, and that, consequently, the mortality was one to 16,000 anæsthesias. (*Westminster Review*, January, 1859.) The error of the calculation, of course, consists in assuming that all the deaths are published. Physicians, resident in Paris, inform me, that only about half of the known deaths from anæsthesia appear in print, in that city, while in England and in this country, probably not one-fifth part are ever published. As a general rule, a surgeon who has killed a patient by chloroform does not feel in a mood for publishing it; hence the whole calculation is worthless, and the idea of the imposing authority of a quarterly review, being lent to the support of such an absurdity, is certainly amusing.

Dr. F. Sabarth, of Wurtemburg, published in French a work which was deemed worthy of translation into German. He estimates that all the anæsthesias in the world, during fourteen years, had amounted to 3,000,000; and finding 119 published cases of deaths, he reckons the mortality as about one in 26,000. It is obvious that this calculation is vitiated by the same blunder of supposing that all the deaths are published.

After the Crimean war, Baudens stated, that there were in that campaign 30,000 chloroformizations, in the French army, without a single death. (*Rev. de deux Mondes*, April, 1857.) Were these figures reliable, they would be a valuable addition to our statistics; but they are under suspicion. In the first place, the round number, 30,000, looks very like a vague estimate, rather than a statistical footing; secondly, a total absence of deaths in 30,000 chloroformed cases is contrary to the experience of all the rest of the world; and, finally, other French surgeons, present in the army, are said to affirm, that they personally knew deaths to occur from chloroform, which were not put on record as such. In the English Crimean army, one chloroform death, at least, is known; but the official reports give us no clue to the number of administrations of the article.

Dr. Richardson, of England, has, perhaps, given more attention to this matter than any European writer. He is quoted by Sansom, as having estimated in the *Medical History of England*, that the ratio of deaths from chloroform is about one in 17,000; but I infer that this is either a misprint, or else Dr. Richardson has since changed his opinion; for he has informed me that he now estimates the mortality about ten times higher than that, *i.e.*, one in 1500. Yet, Sansom's statement is still the basis of opinion for most of the London surgeons, on this subject.

In respect to the dangers of ether, the only information I find is the report of a committee of the Society of Medical Improvement, of Boston, which gives the opinion that no death has ever been known from that agent, while the surgeons of Lyons, in France, who use ether exclusively, and believe it to be much the safest article, yet admit that it occasionally kills in their hands.

The facts collected in the following pages will, I hope, enable us to draw correct conclusions on this subject. The information was mainly obtained by personally visiting the principal hospitals of the world; but it is supplemented by correspondence with spots not visited, as well as by information from other sources, such as reports of the Surgeon-General of the U. S. Army, private records, etc.

In obtaining these facts, my method of procedure was, take from the records of each hospital (where reliable records existed), the number of anæsthesias and the number of resulting deaths. Reports of deaths, not accompanied with the number of anæsthesias, and reports of anæsthesias, not stating the number of deaths, are rigidly rejected. Where reliable records did not exist, I obtained, by personal consultation with the House Surgeons, a careful statement of the annual number of anæsthesias, based on the known average frequency per week, and carried this estimate over any period, during which the same officers could certify positively as to the number of deaths. All these cases are surgical; the results of anæsthesia, as used in midwifery, not coming within the scope of my inquiry.

In deciding what deaths were really caused by the anæsthetics, I have generally followed the opinion of the officers reporting them; but where this could not be obtained, I have adopted the principle, that for a death to be fairly attributed to the anæsthetic, it must be immediate, or nearly so, and there must be no other probable cause present. These rules exclude a great number of deaths, vaguely reported as due to chloroform and ether. Thus, out of 21 reported to the Surgeon-General of the U. S. Army, as caused by chloroform, only seven were found fairly attributable to that agent. It is not possible to keep absolutely clear of all errors, on this point, but I think I have obtained a close approximation to the truth; and as I have pursued exactly the same course with ether as with chloroform, the errors, if any exist, must be fairly distributed on both sides of the question, and the results of the comparison of the two anæsthetics cannot vary much from mathematical verity. The following tables will show the results of the inquiry:—

TABLE OF CHLOROFORMIZATIONS, WITH THE ACCOMPANYING DEATHS.

| SOURCES OF INFORMATION. | CHLOROFORMIZATIONS. | DEATHS. |
|---|---|---|
| Chicago (Hospital and private records), | 6,726 | 5 |
| Bellevue Hospital, N. Y. (about 1867–8), | 600 | 1 |
| Charity " " | 1,460 | 2 |
| Private practice of a Surgeon in Phila., about | 1,000 | 0 |
| U. S. Army Records (Circular No. 6), | 13,956 | 7 |
| Royal Infirmary, Liverpool, | 2,000 | 2 |
| Workhouse Hospital, " | 1,800 | 0 |
| Southern " " | 2,000 | 1 |
| Northern " " | 950 | 0 |
| Charing Cross " London, | 800 | 1 |
| Middlesex " " | 600 | 0 |
| Royal Ophthalmic Hospital, London, | 2,808 | 1 |
| Guy's Hospital (Eye Department)," | 3,224 | 0 |
| " " (Surg. Department)" | 11,500 | 3 |
| *University Hospital, " | 18,250 | 4 |
| Dreadnaught " " | 1,400 | 1 |
| London " " | 13,000 | 3 |
| St. George's " " | 8,000 | 2 |
| Westminster " " | 3,120 | 1 |
| St. Thomas' " " | 3,746 | 2 |
| Other London Hospitals, | 9,826 | 3 |
| La Pitie, Paris, | 312 | 2 |
| K. K. Allg. Kraukenhaus, Vienna, | 10,000 | 2 |
| Totals, | 117,078 | 43 |
| Ratio of Mortality, | | 1 to 2723 |

SULPHURIC ETHER.

In investigating the dangers of ether we are met by contradictory assertions, among those who use it most extensively. A committee of the Boston Society of Medical Improvement, headed by Dr. R. M. Hodges, made a report in 1861, maintaining that no death had yet been correctly attributed to ether. On the other hand, the surgeons of Lyons, in France, who use

* Mr. Erichsen stated, that there were only two deaths, but the House Surgeon informed me that there had positively been four. As Mr. Erichson may have forgotten, or never known of, deaths occurring in the service of the other surgeons, I have adopted the statement of the House Surgeon.

ether exclusively, claim that it occasions a few deaths. I have succeeded in collecting nearly 93,000 cases of anæsthesia, by ether, and among them were found deaths which seem as fairly attributable to that article, as similar cases are to chloroform; though there is a bare possibility of error, of course, in either anæsthetic. Following the rule above indicated, I count, for the present purpose, only the deaths occurring among the 93,000 cases reported to me, and make no assertions *pro* or *con*, about deaths reported outside of that list.

In the New York Hospital, the officers report two deaths from ether, one of which, I reject as not fairly coming within that designation. The rejected case was admitted March, 1864, for a stab in the neck which had penetrated the carotid artery. A large effusion of blood into the tissues occurred, making a tumor which pushed the trachea an inch to the other side, and so pressed upon the nerves as to occasion great pain. Hemorrhage repeated itself about every half hour, till the next day, and was checked each time by pressure of the fingers. The tumor continued to enlarge and press more severely, and the next day it was resolved to make an effort to secure the artery by ligature. The patient was etherized, when the respiration became embarrassed. A second attempt was followed by the same result, and, in a few moments, by death. I conclude, that in this case, the pressure of the tumor on the respiratory nerves was such as to partially paralyze them, requiring the mental attention of the patient to keep up the respiration; and the compression of the trachea might add to the trouble. As soon therefore as the volition was withdrawn by anæsthesia, the respiratory efforts would be embarrassed, and the result become fatal. In fact, the patient might be called already moribund, since the exhaustion of voluntary respiration would bring about the same result in a few hours. In these circumstances, the administration of a full opiate, or any other method of withdrawing the mental attention from the respiratory process, would have a like effect. It may, in a certain sense, be true, that the death occurred a little sooner, on account of the ether; but this is not what we mean by death from the toxic influence of the anæsthetic. I should, without hesitation, reject such a case from the list of deaths by chloroform, and do the same by ether.

The other case was admitted September, 1865, for cancer under the canthus of the eye, which also seemed to occupy the cavities of the maxilla. The patient was etherized, and the operation commenced, everything appearing favorable. No great amount of blood was lost, but, early in the operation, respiration ceased, and the patient died, in spite of all efforts at resuscitation. There was no autopsy. Of course, it is possible that some other cause of death may have been present, but it is not probable that the patient would chance to die at that exact period, from disease, without the ether. Had such a death occurred under chloroform, I should, without hesitation, attribute it to the anæsthetic; and I coincide, therefore, with the officers of the hospital, in calling it a death from ether.

In the hospitals of Lyons, in France, five deaths have been spoken of as caused by ether. These have been carefully discussed by Dr. Gayet, Surgeon-in-Chief of the Hotel Dieu Hospital, of that city.

The first case was in 1852, at the Hotel Dieu, and was precisely like the one in New York Hospital, being a cancer of the superior maxilla. The patient was pale, thin, and cachectic. She was carefully and successfully etherized; but in the early stages of the operation, before the bone was touched, she ceased to breathe, and died, apparently of syncope. It should be mentioned, that the operation was in the sitting posture, a most dangerous position. But little blood was lost, however; and I incline to attribute the syncope to the anæsthetic, and, therefore, rank it as a death from ether.

The second case occurred at the hospital called l'Antiquaille, and was that of a woman etherized, for the removal of vegetations from the vulva. After a time, the pulse was found very feeble, and the operation was abandoned. Efforts to restore her failed, and she died. The surgeon of the hospital considered it a case of death by ether. The autopsy disclosed no cause of death.

The third case was of a man whose leg was crushed off by a locomotive. There were contusions of the abdomen and chest, and great hemorrhage. The shock was great, and the patient already speechless. He was etherized, and the thigh amputated, the patient dying during the operation. This is plainly a death from shock and hemorrhage, and not to be classed with the accidents from anæsthetics.

The fourth case was almost an exact repetition of the third. The patient had a terrible injury of the inferior extremity, with four violent hemorrhages, from a railroad accident. His thigh was amputated, under ether, and he died in less than an hour afterwards. This is evidently a death from shock and hemorrhage.

The last case was of a woman, whose autopsy showed tubercles in the spinal column and in both hip-joints; the cavities of the articulations being filled with cheesy matter, mingled with the debris of the bones. There was also a small tumor in the pia mater of the cerebrum. She was etherized, and the flexed thighs straightened with some force. The adductor muscles, on both sides, were torn, and the neck of one femur broken, by the operation. There was also considerable blood effused. The patient showed signs of syncope, but, by the prompt use of restoratives, recovered her pulse and color, and was carried to her bed by the attendants. Arriving at the bed, she fainted a second time; and the nurses not understanding what to do, she was past recovery before the surgeon was called. There is more doubt about this case than about either of the others; but after considering the terrible condition, both of the brain, the spine, and the hip-joints, and the natural results of extensive laceration of muscles, effusion of blood, and fracture of the neck of the femur on such an exhausted constitution, it appears to me that the ether was not chargeable with the death. I coincide with the officers of the hospital, therefore, in the opinion that the patient succumbed to simple syncope, caused by the operation, and rendered fatal by the accidental absence of skilled assistance, on its second recurrence. Two other cases of death have been spoken of, in the vicinity of Lyons, as caused by ether; but not being in the hospitals, they do not come within the scope of my statistics.

Finally, one case of death by ether is reported to me verbally, by the officers of St. Thomas' Hospital, London. I could not learn the particulars.

We have, therefore, four deaths, apparently caused by ether, in circumstances where we can make a comparison with the total number of cases of anæsthesia, among which they occurred. The following table gives a view of the results:—

TABLE OF ETHERIZATIONS WITH THE ACCOMPANYING DEATHS.

| SOURCES OF INFORMATION. | ETHERIZATIONS. | DEATHS. |
|---|---|---|
| Chicago Records, | 895 | 0 |
| Mass. General Hospital, Boston, about | 25,000 | 0 |
| City Hospital, " " | 8,760 | 0 |
| New York Hospital, New York, | 5,100 | 1 |
| Bellevue " " (about 1868–69), | 600 | 0 |
| Pennsylvania " Philadelphia, | 2,500 | 0 |
| Episcopal " " | 3,432 | 0 |
| Private Practice, " | 250 | 0 |
| U. S. Army (Circular No. 6.), | 6,978 | 0 |
| St. Thomas' Hospital, London, about | 1,000 | 1 |
| LaPitie " Paris, about | 300 | 0 |
| Hospitals of Lyons, about | 38,000 | 2 |
| Totals, | 92,815 | 4 |
| Ratio of Mortality, | 1 to 23,204 | |

It is evident, therefore, that the ratio of mortality, in surgical anæsthesia, by chloroform, is about eight and a-half times greater than by ether.

Sufficient statistics upon the subject of mixtures of chloroform and ether have not been collected to form a sure basis of opinion. The following table, so far as it goes, seems to show that the ratio of danger is, as might be expected, between the other two:—

TABLE OF ANÆSTHESIAS, BY MIXTURES OF CHLOROFORM AND ETHER, WITH THE ACCOMPANYING DEATHS.

| Sources of Information. | Anæsthesias. | Deaths. |
|---|---|---|
| Chicago Statistics, about | 350 | 1 |
| U. S. Army (Circular No. 6), | 2,326 | 0 |
| Guy's Hospital, London | 8,500 | 1 |
| Totals, | 11,176 | 2 |
| Ratio of Mortality, | 1 to 5588 | |

From this it would seem that the mixtures have proved twice as safe as chloroform, and about four times more dangerous than ether.

With regard to the new anæsthetic, bichloride of methylene, we have as yet no decisive results, but the English journals report a single death among about 7000 cases.

Nitrous oxide, contrary to our first impressions, turns out to be the safest of all anæsthetics. It is not easy to place the result in figures; but in cities where we constantly have deaths from chloroform, we seldom hear of any by the gas. The only statistics which I know of on the subject are those kept by the Colton Dental Association. This is an associated circle of dental establishments, with branches in all the largest cities, which confine themselves exclusively to the extraction of teeth, and invariably use the gas as an anæsthetic, and preserve a record of each case. The plan of this record was prepared by Dr. Colton, at the suggestion of a member of Congress. I have no means of positively verifying its accuracy, but, yet, I attribute considerable value to it. The Association, at the present time, claims to have anæsthetized about 75,000 patients, in the United States, without any death.

Finally, if we summarize the whole matter, it seems that the various anæsthetics have the following rates of mortality:—

| | | | | | |
|---|---|---|---|---|---|
| Sul. Ether, | 1 death to | | | 23,204 | administrations. |
| Chloroform, | 1 | " | " | 2,723 | " |
| Mixed Chloroform and Ether, | 1 | " | " | 5,588 | " |
| Bichloride of Methylene, | 1 | " | " | 7,000 | " |
| Nitrous Oxide, | No death in | | | 75,000 | " |

